# Bexarotene inhibits the viability of non-small cell lung cancer cells via slc10a2/PPARγ/PTEN/mTOR signaling pathway

**DOI:** 10.1186/s12885-018-4224-x

**Published:** 2018-04-11

**Authors:** Xinghao Ai, Feng Mao, Shengping Shen, Yang Shentu, Jiejun Wang, Shun Lu

**Affiliations:** 1 Department of Medical Oncology, Changzheng Hospital, The Second Military Medical University, Shanghai, 200433 China; 2 Lung Tumor Clinical Medical Center, Shanghai Chest Hospital, Shanghai Jiao Tong University, Shanghai, 200030 China

**Keywords:** Non-small cell lung cancer, A549 cells, H1299 cells, Bexarotene, slc10a2, PPARγ

## Abstract

**Background:**

Thirty to 40 % of non-small cell lung cancer (NSCLC) patients developed higher hypertriglyceridemia in the process of treatment with bexarotene. And bioinformatics studies discovered that the expression of slc10a2 was increased in high-grade hypertriglyceridemia patients. So, we will explore the mechanism which may involve in this process.

**Methods:**

We constructed slc10a2 overexpressed A549 cells and H1299 cells as cell models, normal A549 cells and H1299 cells as control. Then we explored the effects of slc10a2 on A549 cells and H1299 cells behaviors, including proliferation, invasion and apoptosis. The expression of apoptotic related genes and anti-cancer genes also been detected.

**Results:**

We found that the proliferation and migration were inhibited and the apoptosis of NSCLC cells was accelerated by bexarotene. In addition, overexpressed slc10a2 in NSCLC cells can further suppress the proliferation and migration, and promote apoptosis under the treatment of bexarotene. On the contrary, the opposite results were obtained after slc10a2 gene was silenced in NSCLC cells treated with bexarotene. Moreover, the expression of caspase 3, caspase 7, PTEN, P21, P53, LKB1, TSC2 were increased and the expression of Bcl-2, cyclin D1, c-FLIP were declined in NSCLC cells and slc10a2 overexpressed NSCLC cells with the treatment of bexarotene, and the opposite situations were seen after slc10a2 gene was silenced in NSCLC cells. The further studies revealed the increased expression of slc10a2 activated the expression of peroxisome proliferator-activated receptor γ (PPARγ), then up-regulated PTEN expression and down-regulated mTOR expression.

**Conclusion:**

These results suggest that bexarotene inhibits the viability of lung cancer cells via slc10a2/PPARγ/PTEN/mTOR signaling pathway.

**Electronic supplementary material:**

The online version of this article (10.1186/s12885-018-4224-x) contains supplementary material, which is available to authorized users.

## Background

The incidence of lung cancer is rapidly increasing in the world, and it has become the first leading cause of cancer death, especially in China [1]. Non-small cell lung cancer (NSCLC) is the most common type of lung cancer, accounting for almost 80% [2]. In clinic trials, bexarotene showed both satisfactory safety and promising efficacy for the treatment of advanced NSCLC patients [3, 4]. However 30–40% of the patients appeared to be more sensitive to bexarotene treatment and developed higher hypertriglyceridemia. Interestingly, survival analysis in high-grade hypertriglyceridemia patients revealed significantly longer survival compared to the patients in the control, low-grade hypertriglyceridemia and middle-grade hypertriglyceridemia groups [5, 6].

**Scheme 1:**
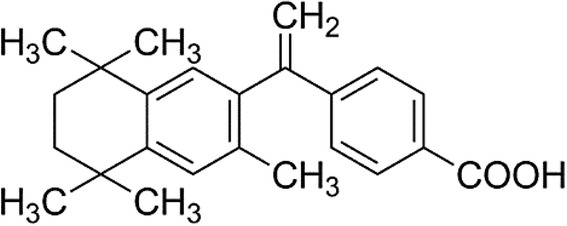
The chemical structure of bexarotene

Bexarotene (Scheme 1) is a synthetic retinoid modulator of retinoid X receptors (RXRs), it can selectively bind and activate RXRs [2], which include (RXRα, RXRβ, and RXRγ) [7], and play a critical role in cellular growth modulation, activation of apoptosis, induction of differentiation. It has been widely explored as potential target for cancer therapies for several years [8, 9]. The expression of RXRs was reduced in some NSCLC biopsy specimens, and increased RXRs expression has been associated with an increased survival in NSCLC patients [10].

Slc10a2 is a member of solute carrier family 10 of the sodium/bile acid co-transporter apical sodium-dependent bile acid transporter (ABST) [11], which plays a key role in the enterohepatic circulation through its reabsorption of bile acids from the ileum and indirectly conduces to cholesterol homoeostasis [12, 13]. ASBT is able to inhibit the concentration of plasma triglyceride and increase the concentration of HDL (high-density lipoprotein) cholesterol [14], and now it has aroused much concern as a drug target for the pharmacological treatment of hypercholesterolaemia [15, 16].

The goal of this study is to explore the role of slc10a2 in the treatment of NSCLC with bexarotene. We hypothesis that bexarotene inhibits the viability of NSCLC cells (e.g. A549 cells and H1299 cells) via increasing the expression of slc10a2. In this study, we have successfully constructed slc10a2 overexpressed A549 cells and H1299 cells, and the proliferation, apoptosis, migration behaviors were detected in slc10a2 overexpressed A549 cells and H1299 cells respectively. Moreover, we also explored the expression of apoptosis genes, anti-apoptosis genes, tumor suppressor genes in slc10a2 overexpressed A549 cells and H1299 cells. Furthermore, the possible mechanism which involved in this process was discovered.

## Methods

### Materials

Bexarotene was obtained from Aladdin (Shanghai, China). Cell Counting Kit-8 was ordered from Dojindo (Japan). Cell culture plates and Transwell plates were ordered from Corning (NY, USA). Crystal violet was obtained from Beyotime (Haimen, China). Annexin V/fluorescein isothiocyanate (FITC) apoptosis detection kit was obtained from Beyotime biotech company (China). Gentamicin, Fetal bovine serum, glutamine, and RPMI 1640 medium were purchased from Thermo Fisher Scientific (Waltham, MA, USA). The primary antibodies including slc10a2, PPARγ, mTOR and PTEN (Abcam, Cambridge, UK), GAPDH (Thermo, Walteham, Washington, USA). The pcDNA3 and pcDNA3-slc10a2 plasmid, slc10a2-shRNA, GW9662 were obtained from Shanghai Funeng Biological Technology, Co., LTD.

### Cell lines

The human NSCLC cell lines A549 cells (CRM-CCL-185™) and H1299 cells (CRL-5803™) was obtained from American Type Culture Collection (Rockville, MD). Cells were maintained in RPMI 1640 media plus 10% fetal bovine serum, 1% glutamine and 0.05 mg/ml gentamycin sulfate at 37 °C and 5% CO_2_.

### The construction of slc10a2 overexpressed A549 cells and H1299 cells

Before transfection, A549 cells and H1299 cells were seeded into 6-well plate at a density of 2 × 10^6^ cells/well, after cells grow above 80% areas, A549 cells were transfected with 8 mg pcDNA3-slc10a2 plasmid using Lipofect2000 transfection Reagent according to manufacturer’s instructions, pcDNA3 treatment as control. The transfection medium was replaced by regular growth medium after 5 h transfection, and at each time point, the cells were used to observation using inverted fluorescence microscope.

### Proliferation assay

A549 cells, H1299 cells and slc10a2 overexpressed A549 cells, H1299 cells were seeded in a 96-well plate, 5 × 10^3^ cells per well. After 12 h culture, the cultured medium was replaced by conditional medium, which was added with bexarotene, bexarotene in combination with slc10a2-shRNA, bexarotene in combination with GW9662 respectively. At indicated time point, ten microliter CCK-8 was added each well and continually incubated for 4 h. Then the optical density (OD) value (450 nm) was determined by an enzyme-linked immunosorbent assay plate reader (Bioreader).

### Transwell migration assay

A549 cells and slc10a2 overexpressed A549 cells were starved for 12 h, then resuspended in serum-free medium, and adjusted to 1 × 10^6^ cells/ml. One hundred microliters of A549cells or slc10a2 overexpressed A549 cells were placed in the upper chamber of Transwell plates. Serum-free RPMI 1640 medium with bexarotene, bexarotene in combination with slc10a2-shRNA, was added to the lower chamber respectively, RPMI 1640 medium as control. Prior to the addition of cells suspension, preheated serum free RPMI 1640 medium (300 μl) was added to the upper chamber. After 24 h incubation, the invaded cells were collected from lower chambers, then stained with crystal violet.

### Apoptosis assay

The apoptosis of A549 cells, H1299 cells and slc10a2 overexpressed A549 cells, H1299 cells was analyzed using the Apoptosis Detection Kit according to the manufacturer’s instructions. Cells were seeded in 6-well plate (1 × 10^5^ cells/well) with different medium, including RPMI 1640 medium plus bexarotene, RPMI 1640 medium plus bexarotene in combination with slc10a2-shRNA for 2 days. At indicated times, cells were digested then resuspended in 300 μL binding buffer solution which containing 5 ul Annexin V-FITC and 5 ul PI solution, then incubated in the dark for 20 min at room temperature. Finally flow cytometry (FACScan; BD Biosciences) was used to analyzed the apoptotic rate of each kind of cells.

### RT quantitative-PCR analysis

The total RNA of A549 cells and H1299 cells were isolated by using RNeasy kit according to the manufacturer’s protocol (Qiagen, Valencia, CA). Briefly, total RNA (1 μg) was used as a template to prepare cDNA (Invitrogen), and was amplified by Platinum SYBR Green qPCR SuperMix-UDG (Invitrogen). A master mix was prepared for each PCR reaction, which included Platinum SYBR Green qPCR SuperMix-UDG, forward primer, reverse primer, and 10 ng of template cDNA. PCR was performed with the following thermocycling conditions: An initial 5 min at 95 °C, followed by 40 cycles of 95 °C for 30 s, 55 °C for 30 s and 72 °C for 30 s. [17] The forward and backward primer sequences for Bcl-2 was 5’-CCGATCAGTGGAGCTGAAGAA-3′(sense) and 5’-GCCACAGGATGTTCTCGTCA-3′(antisense), cyclinD1:5’-CAAGGCCTGAACCTGAGGAG-3′(sense) and 5’-CTTGGGGTCCATGTTCTGCT-3′(antisense), c-FLIP:5’-GAGTGCCGGCTATTGGACTT-3′(sense) and 5’-GCGCTTCTCTCCTACACCTC-3′(antisense), Caspase-3:5’-GCGGTTGTAGAAGTTAATAAAGGT-3′(sense) and 5’-TACCAGACCGAGATGTCATTCC-3′(antisense), Caspase-7:5’-CGTGGGAACGGCAGGAAGT-3′(sense) and 5’-CGGGTGGTCTTGATGGATCG-3′(antisense), PTEN:5’-CAGGATACGCGCTCGGC-3′(sense) and 5’-TCAGGAGAAGCCGAGGAAGA-3′(antisense), P21:5’-AGTCAGTTCCTTGTGGAGCC-3′(sense) and 5’-CATTAGCGCATCACAGTCGC-3′(antisense), P53:5’-GTGCTCAAGACTGGCGCTAAA-3′(sense) and 5’-CAGTCTGGCCAATCCAGGGAAG-3′(antisense), LKB1:5’-GACCTGCTGAAAGGGATGCT-3′(sense) and 5’-GACCTGCTGAAAGGGATGCT-3′(antisense), TSC2:5’-TCTGAACATGTGGTCCGCAG-3′(sense) and 5’-TCTGAACATGTGGTCCGCAG-3′(antisense).

### Western blot

A549 cells and H1299 cells were treated with 1 mM, 5 mM, 10 mM bexarotene for 24 h, then the cells were harvested, then total protein from tissue or cell was extracted using radioimmunoprecipitation lysis buffer containing 1 mM phenylmethanesulfonylfluoride and the protein concentration was determined using the Bradford method (Beyotime Institute of Biotechnology, Nantong, China) according to the manufacturer’s instructions. Proteins (20 μg) were separated by 10% SDS-PAGE and transferred onto a nitrocellulose membrane. After blocking with 5% non-fat milk for 1 h at 4 °C, then membrane was incubated with the primary antibody at 4 °C overnight. Membranes were washed 3 times with 0.25% PBST and then incubated with the peroxidase-conjugated secondary antibody for 2 h at room temperature. After washed 3 times, the specific protein bands were detected using the enhanced chemiluminescence reagents [18].

### Statistical analysis

All data were expressed as mean ± S.D. Differences between the groups were analyzed using one-way analysis of variance (ANOVA) using SPSS 13.0 (SPSS Inc, Chicago, IL, USA). *p*-values less than 0.05 were considered statistically significant.

## Results

### The construction of slc10a2 overexpressed NSCLC cells

**Fig. 1:**
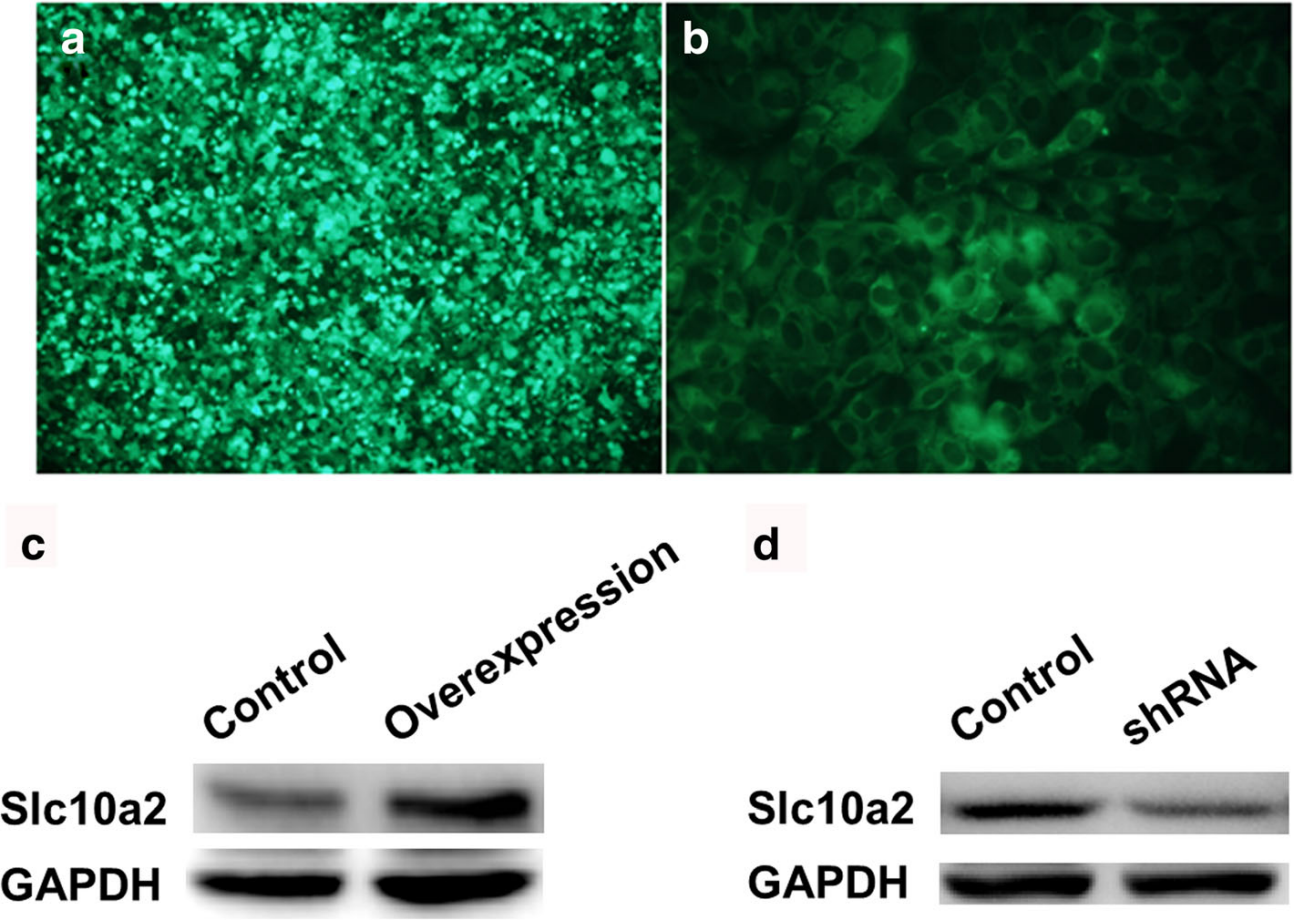
The construction and identification of slc10a2 overexpressed A549 cells. **a** The immunofluorescence staining showed slc10a2 gene had successfully transfected into 293 T cells; **b** The immunofluorescence staining showed slc10a2 overexpressed A549 cells were successfully constructed; **c** The expression of slc10a2 was significant higher in slc10a2 overexpressed A549 cells than in A549 cells; **d** Slc10a2-shRNA can effectively inhibit the expression of slc10a2 in A549 cells. All experiments were repeated 3 times

As shown in Fig. 1a, after 24 h treatment with pcDNA3.1-slc10a2 plasmid in 293 T cells, the 293 T cells had successfully transfected with slc10a2 gene, and efficiency of transfection is about 92%. Then the supernatant of virus was added to A549 cells, after this slc10a2 overexpressed A549 cells were successfully constructed (Fig. 1b). Similarly we also used this method to construct slc10a2 overexpressed H1299 cells (data not show). The western blot results further demonstrated that the expression of slc10a2 was obviously higher in transfected group than control group (Fig. 1c). And the expression of slc10a2 was declined after treated with slc10a2-shRNA in A549 cells, this result suggests slc10a2-shRNA can effectively prohibit the expression of slc10a2 in A549 cells (Fig. 1d). In addition, slc10a2-shRNA also can significantly inhibit the expression of slc10a2 in H1299 cells (data not show).

### Slc10a2 plays an important role in the proliferation of NSCLC cells with the treatment of bexarotene

**Fig. 2:**
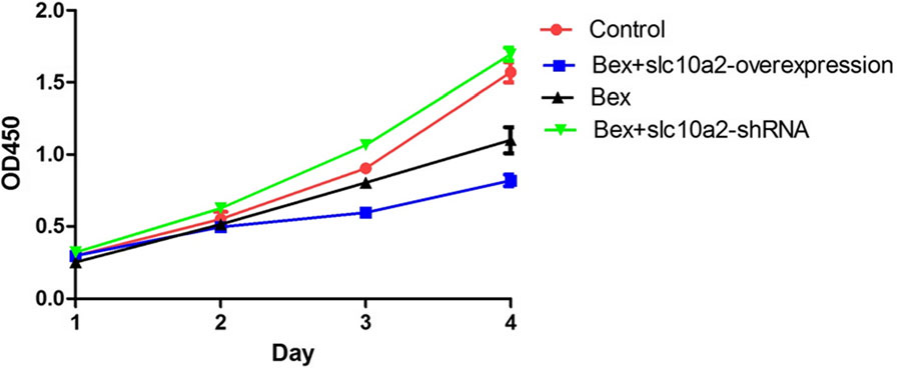
The proliferation of A549 cells treated with bexarotene, bexarotene + slc10a2-shRNA, bexarotene + slc10a2-overexpression respectively. A549 cells without treatment as control. All experiments were repeated 3 times

As Fig. 2 showed, in comparison to the control group (without any treatment), bexarotene can inhibit the proliferation of A549 cells on day 3 and day 4. And the proliferation of slc10a2 overexpressed A549 cells was significantly prohibited with the treatment of bexarotene on both day 3 and day 4. However, the proliferation of A549 cells was increased when co-treated with bexarotene and slc10a2-shRNA at all of the detected time points. Moreover, the same results can be seen in H1299 cells (Additional file 1: Figure S1A). These results suggest slc10a2 involve in the process of bexarotene inhibits the proliferation of NSCLC cells.

### Slc10a2 plays an important role in the invasion of NSCLC cells with the treatment of bexarotene

**Fig. 3:**
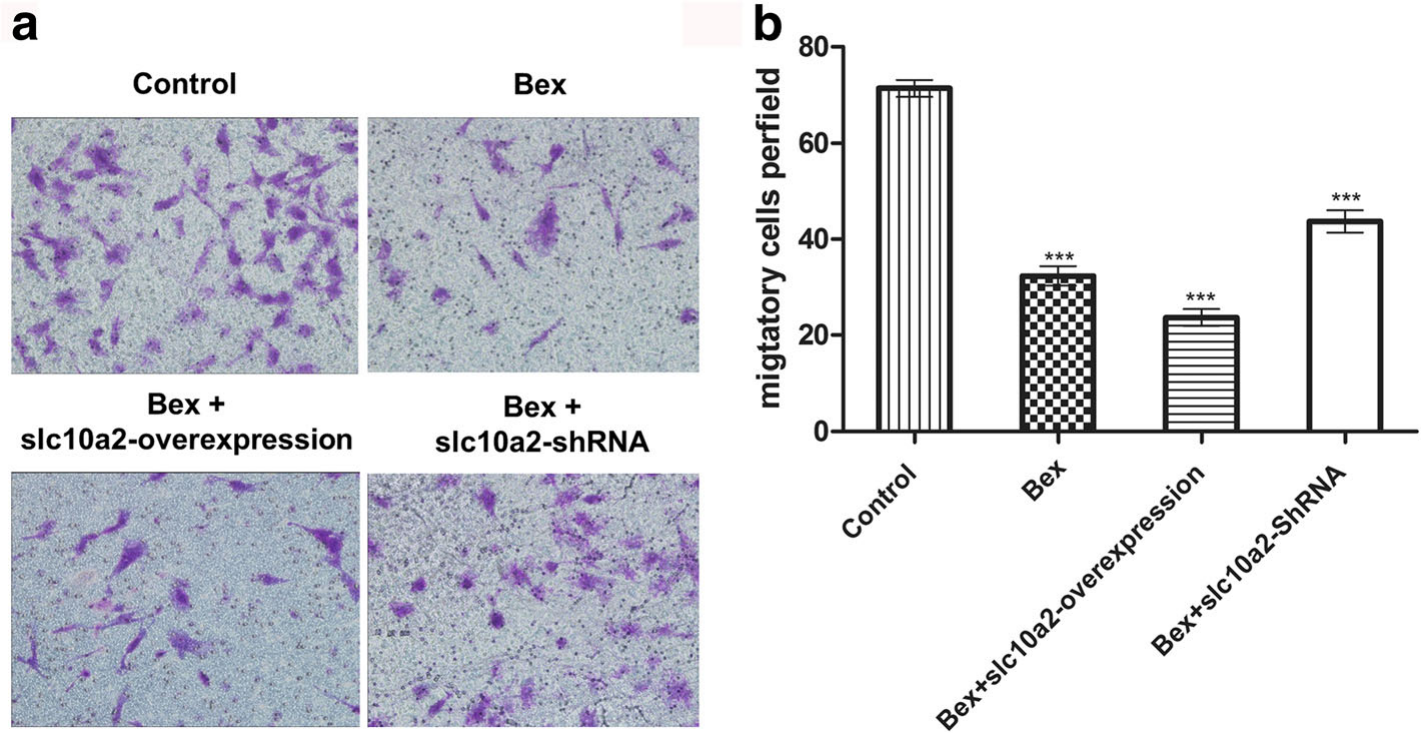
The effects of slc10a2 on invasion of A549 cells treated with bexarotene. **a** The invasion behavior of A549 cells treated with bexarotene, bexarotene + slc10a2-shRNA, bexarotene + slc10a2-overexpression respectively, A549 cells without any treatment as control group. **b** The quantification of migratory A549 cells treated with bexarotene, bexarotene + slc10a2-shRNA, bexarotene + slc10a2-overexpression respectively. All experiments were repeated 3 times. ***p < 0.001

Transwell invasion test showed that (Fig. 3), the migration of A549 cells was decreased with the treatment of bexarotene for 24 h when compared with control group, the similar situation was discovered in slc10a2 overexpressed A549 cells treated with bexarotene. While the migration of A549 cells was increased when co-treated with bexarotene and slc10a2-shRNA. It reveals that slc10a2 involve in the process of bexarotene inhibits the invasion of NSCLC cells.

### Slc10a2 plays an important role in the apoptosis of NSCLC cells with the treatment of bexarotene

**Fig. 4:**
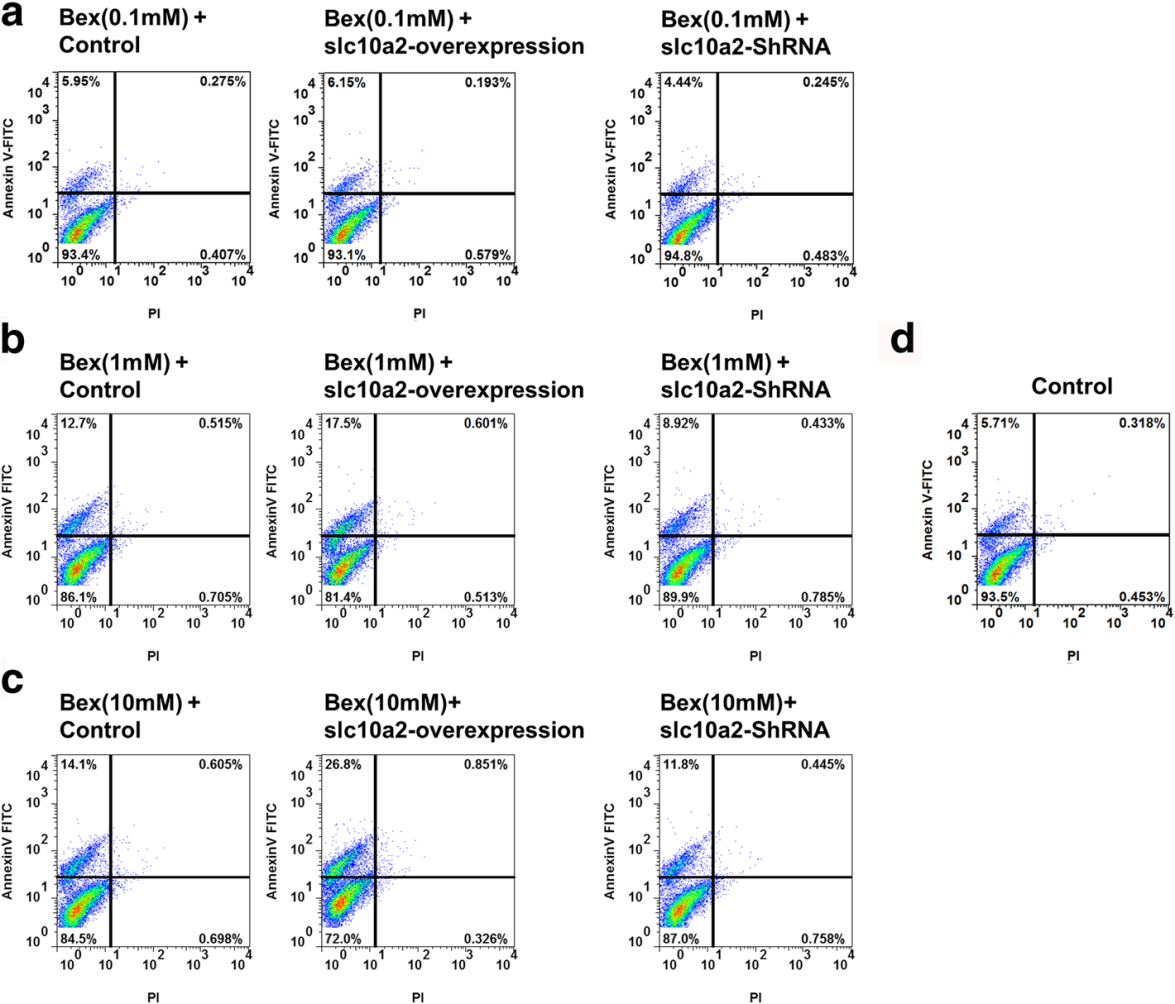
The effects of slc10a2 on apoptosis of A549 cells treated with bexarotene. **a** The apoptosis rate of A549 cells treated with 0.1 mM bexarotene, overexpressed slc10a2 in combination with 0.1 mM bexarotene, slc10a2-shRNA in combination with 0.1 mM bexarotene respectively; **b** The apoptosis rate of A549 cells treated with 1 mM bexarotene, overexpressed slc10a2 in combination with 1 mM bexarotene, slc10a2-shRNA in combination with 1 mM bexarotene respectively; **c** The apoptosis rate of A549 cells treated with 10 mM bexarotene, overexpressed slc10a2 in combination with 10 mM bexarotene, slc10a2-shRNA in combination with 10 mM bexarotene respectively, A549 cells without any treatment as control group (**d**). All experiments were repeated 3 times

As shown in Fig. 4a, in comparison to the control group (Fig. 4d**)**, there is no significant difference in apoptosis rate between 0.1 mM bexarotene treated group and control group. However, the apoptosis rate in 1 mM and 10 mM bexarotene treated groups was obviously higher than control group. In addition, the apoptosis rate of slc10a2 overexpressed A549 cells treated with 1 mM and 10 mM bexarotene was significantly increased than in A549 cells, and it distinctly declined in A549 cells after treated with slc10a2-shRNA in combination with bexarotene when compared to the bexarotene single treated group (Fig. 4b and c). Moreover, the apoptosis of H1299 cells was significant increased when H1299 cells treated with 10 mM bexarotene + slc10a2-overexpression, and it decreased when H1299 cells treated with 10 mM bexarotene + shRNA-slc10a2 (Additional file 1: Figure S1B).

### Slc10a2 plays an important role in tumor suppressor with the treatment of bexarotene

**Fig. 5:**
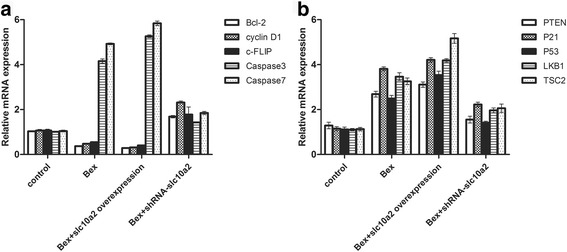
The effects of slc10a2 on expression of apoptosis related genes in A549 cells treated with bexarotene. **a** The expression of apoptotic related genes Bcl-2, cyclin D1, c-FLIP, caspase 3, caspase 7 in A549 cells treated with bexarotene, overexpressed slc10a2 in combination with bexarotene, slc10a2-shRNA in combination with bexarotene respectively, A549 cells without any treatment as control group. **b** The expression of tumor suppressor genes PTEN, P21, P53, LKB1, TSC2 in A549 cells treated with bexarotene, overexpressed slc10a2 in combination with bexarotene, slc10a2-shRNA in combination with bexarotene respectively, A549 cells without any treatment as control group. All experiments were repeated 3 times

As shown in Fig. 5 and Additional file 2: Figure S2, after A549 cells and H1299 cells treated with bexarotene for 24 h, the expression of apoptotic related genes caspase 3, caspase 7, and tumor suppressor genes PTEN, P21, P53, LKB1 and TSC2 were significantly increased (*p* < 0.05) and the expression of anti-apoptotic genes bcl 2, cyclin D1, c-FLIP were decreased (*p* < 0.05). Similarly, the same situations were observed in slc10a2 overexpressed A549 cells and H1299 cells. While, the expression of caspase 3, caspase 7, PTEN, P21, P53, LKB1 and TSC2 were reduced (*p* < 0.05) and the expression of bcl 2, cyclin D1, c-FLIP were increased (*p* < 0.05) when A549 cells and H1299 cells were co-treated with bexarotene and slc10a2-shRNA.

### Slc10a2 via PPARγ plays an important role in the proliferation of NSCLC cells with the treatment of bexarotene

**Fig. 6:**
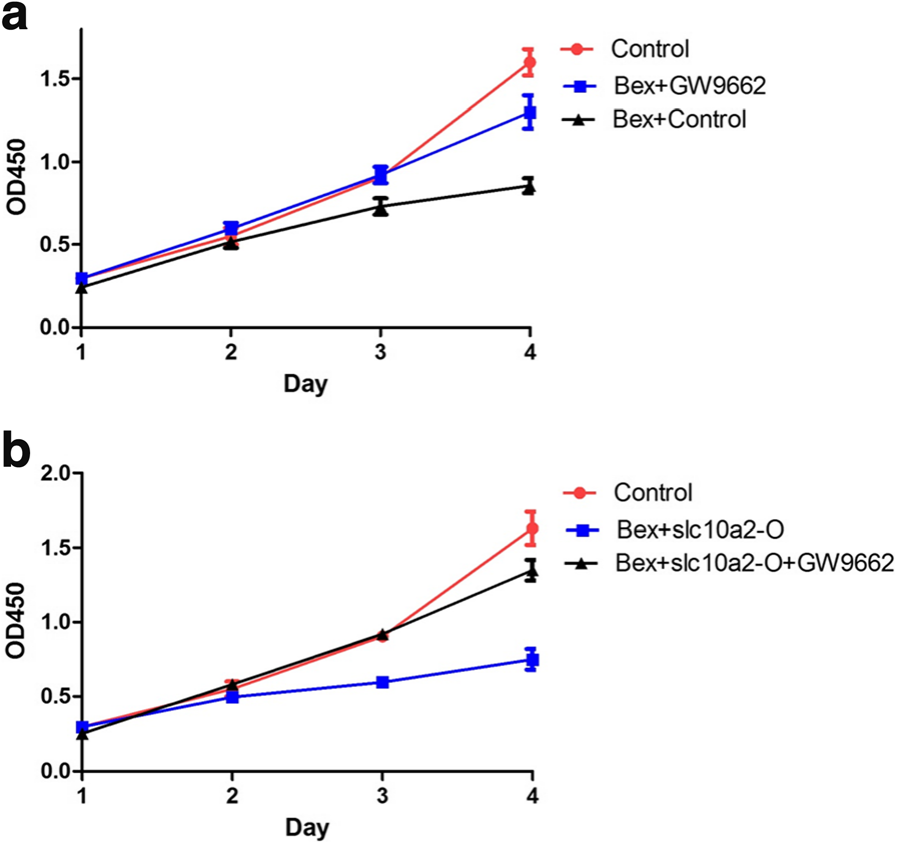
Slc10a2 via PPARγ plays an important role in the proliferation of A549 cells with the treatment of bexarotene. **a** The proliferation rate of A549 cells treated with bexarotene, bexarotene in combination with GW9662 respectively, A549 cells without any treatment as control group. **b** The proliferation rate of slc10a2 overexpressed A549 cells treated with bexarotene, bexarotene in combination with GW9662 respectively, A549 cells without any treatment as control group. All experiments were repeated 3 times

According to the aforementioned, we have demonstrated that slc10a2 plays an important role in the proliferation of A549 cells with the treatment of bexarotene, how can the slc10a2 effect in this process. As Fig. 6a showed, by comparison, the proliferation of A549 cells was inhibited when treated with bexarotene on both day 3 and day 4. However, we found that GW992 (selective PPARγ antagonist) can shortened the proliferative inhibition effects of bexarotene. The proliferation rate of A549 cells was higher in bexarotene in combination with GW9662 treated group than the bexarotene treated group on day 3 and day 4. Furthermore, a similar result was found in slc10a2 overexpressed A549 cells (Fig. 6b).

### Slc10a2 via PPARγ plays an important role in tumor suppressor with the treatment of bexaroten

**Fig. 7:**
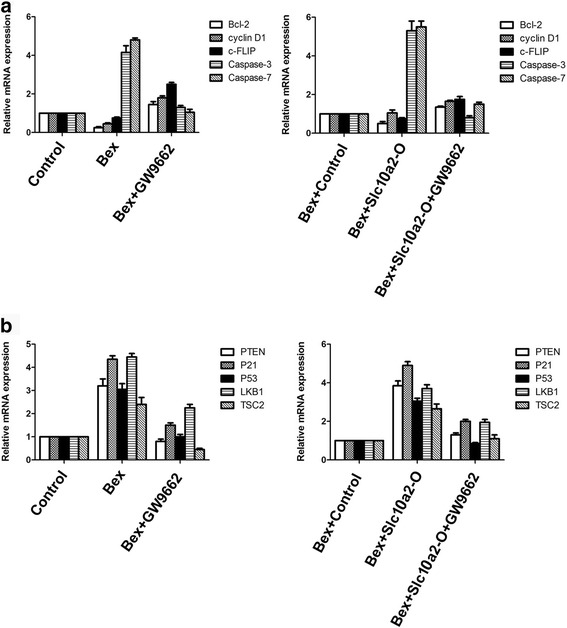
Slc10a2 via PPARγ plays an important role in tumor suppressor with the treatment of bexarotene. **a** The expression of apoptotic related genes Bcl-2, cyclin D1, c-FLIP, caspase 3, caspase 7 in A549 cells and slc10a2 overexpressed A549 cells when treated with bexarotene, bexarotene in combination with GW9662 respectively; **b** The expression of tumor suppressor genes PTEN, P21, P53, LKB1, TSC2 in A549 cells and slc10a2 overexpressed A549 cells when treated with bexarotene, bexarotene in combination with GW9662 respectively. A549 cells without any treatment as control group. All experiments were repeated 3 times

We further explored whether slc10a2 via PPARγ plays an important role in tumor suppressor with the treatment of bexarotene. As shown in Fig. 7 and Additional file 3: Figure S3 after A549 cells, H1299 cells or slc10a2 overexpressed A549 cells, H1299 cells treated with bexarotene, the expression of apoptotic genes caspase 3, caspase 7, and tumor suppressor genes PTEN, P21, P53, LKB1 and TSC2 were significantly increased (*p* < 0.05) and the expression of anti-apoptotic genes bcl 2, cyclin D1, c-FLIP were reduced (*p* < 0.05). While, the expression of caspase 3, caspase 7, PTEN, P21, P53, LKB1 and TSC2 were declined (*p* < 0.05) and the expression of bcl 2, cyclin D1, c-FLIP were increased (*p* < 0.05) when A549 cells, H1299 cells or slc10a2 overexpressed A549 cells, H1299 cells were co-treated with bexarotene and GW9662.

### Bexarotene inhibits the viability of NSCLC cells via slc10a2/PPARγ/PTEN/mTOR signaling pathway

**Fig. 8:**
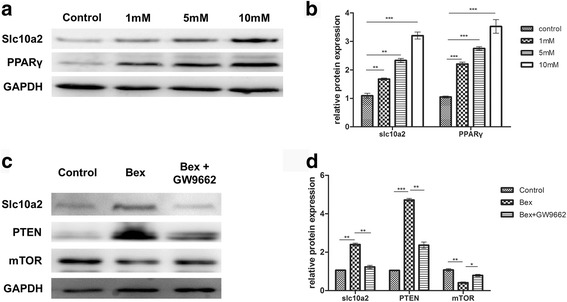
Bexarotene inhibits the viability of A549 cells via slc10a2/PPARγ/PTEN/mTOR signaling pathway. **a** The expression of slc10a2 and PPARγ in A549 cells treated with 1 mM, 5 mM, 10 mM bexarotene respectively. **b** The quantification of slc10a2 and PPARγ expression in A549 cells treated with 1 mM, 5 mM, 10 mM bexarotene respectively. **c** The expression of slc10a2, PTEN, mTOR in A549 cells treated with bexarotene, bexarotene + GW9662 respectively. **d** The quantification of slc10a2, PTEN, mTOR expression in A549 cells treated with bexarotene, bexarotene + GW9662. All experiments were repeated 3 times. *p < 0.05, **p < 0.01

As shown in Fig. 8a, b and Additional file 4: Figure S4, the western blotting and RT-PCR results showed the expression of slc10a2 was gradually enhanced with the increase of bexarotene’s concentrations from 1 mM to 10 mM. Also the expression of PPARγ was increased with the increase of bexarotene’s concentrations. Additionally, the expression of slc10a2 can be reduced in Bex + GW9662 treated group. Moreover the expression of PTEN was increased in bexarotene treated group when compared to the control group, while it can be inhibit when A549 cells treated with bexarotene and GW9662. On the contrary, the expression of mTOR was suppressed by bexarotene, and this inhibition effects can be shortened by GW9662 (Fig. 8c, d).

## Discussion

Three types of PPARs have been identified: alpha, gamma, and delta, they are a group of nuclear receptor proteins which function as transcription factors regulating the expression of genes [19]. Among the three phenotypes, PPARγ has been attracting tremendous attention, the previous studies revealed that PPARγ plays essential roles in the regulation of cellular differentiation, development, and metabolism (carbohydrate, lipid, protein), and tumorigenesis [20, 21].

A mass of studies have demonstrated that PPARγ agonists via inhibiting the expression of cyclinD1, cyclinB, cyclinE, CDK4 and CDK2 and increasing the expression of CDKN1A to prevent cell cycle from G1 to S phase to prohibit tumor cells proliferation [22–26]. In this study we found that bexarotene worked as a PPARγ agonists, which was capable of enhancing the expression of PPARγ, then the expression of cyclinD1 was suppressed and the proliferation of A549 cells was prohibited, these results were consistent with the previous studies.

The activation of PPARγ can induce tumor cell apoptosis via several different pathways. PPARγ agonists was able to up-regulate pro-apoptotic protein BAX and BAD expression, and then induced glioma cells apoptosis through releasing cytochrome C and activating the activation of caspase [27]. Li et al. reported the activation of PPARγ was associated with a decrease of the expression of Bcl-2 and increase of the expression of P53 in human melanoma cell line A375 cells [28]. Similarly, we found that the expression of anti-apoptotic proteins Bcl-2, cyclin D1 and c-FLIP was reduced whereas the expression of apoptotic proteins caspase-3, caspase-7 and tumor suppressor gene PTEN, P21, P53, LKB1, TSC2 were accelerated in A549 cells with the treatment of bexarotene, which was associated with the activation of PPARγ through enhancing the expression of slc10a2, resulting in promoting the apoptosis of A549 cells.

Moreover, the activation of PPARγ can reduce the invasion ability of tumor cells. Thiazolidinedione (TZD) is a synthetic agonist of PPARγ, which contains troglitazone, pioglitazone and rosiglitazone [29]. Willson et al. discovered that after adrenocortical cancer cell lines H295R cells co-cultured with pioglitazone and rosiglitazone, the expression of MMP-2 which play an important role in cell migration was reduced, and the migration of H295R cells was significantly declined [30]. Also Galli et al. found that TZD can effectively inhibit tumor cell invasion, after pancreatic cancer cell treated with TZD for 24 h, the activity and transcriptional level of MMP-2 were declined [31]. And in this study, the migration ability of A549 cells was significantly shortened after treated with bexarotene, whereas the migration of A549 cells was distinctly accelerated after with the treatment of bexarotene in combination with GW9662.

PI3K/Akt/mTOR signaling pathway exists in almost all mammals, it regulates cell growth mainly through controlling the protein synthesis. PTEN as a negative regulator of this pathway via suppressing the expression of PI3K and Akt. Patel et al. revealed that PPARγ can combined with peroxisome proliferator responsive element 1 (PPRE1) and PPRE2, the upstream gene of PTEN, and increased the expression of PTEN then induced phosphorylation of Akt decreased, cells differentiation and apoptosis [32]. And it has been preclinically showed that deficiency of TSC2 or PTEN expression induces impaired PI3K/Akt/mTOR activation, suggesting that mTOR overexpression with the loss of PTEN plays a key role in the development and progression of pancreatic neuroendocrine tumors [33]. In this study, we discovered that bexarotene accelerated the expression of PPARγ through enhancing the expression of slc10a2, also the expression of PPARγ was promoted, while the expression of mTOR was declined, thus the viability of A549 cells was suppressed. Finally this study has some limitations, for example, PPAR agonist bexarotene induces PPARγ and slc10a2 in a dose dependent manner, but the effects of bexarotene on PPARα, PPARβ/δ expression we don’t explore. We have clarified limitations in the discussion section, and we will explore the effects of bexarotene on PPARα, PPARβ/δ expression in our future study.

## Conclusion

In this study we found that bexarotene can suppress the proliferation, migration, and promote the apoptosis of NSCLC cells. Moreover, we demonstrated that this effects owned to the increased expression of PPARγ via enhancing the expression of slc10a2, then up-regulated the expression of PTEN and down-regulated the expression of mTOR, thus increased the expression of apoptotic genes and anti-cancer genes, and reduced the expression of anti-apoptotic genes to suppress the proliferation of NSCLC cells and promote the apoptosis of NSCLC cells [34].

## Additional files

Additional file 1: **Figure S1.** (A) The proliferation of H1299 cells treated with bexarotene, bexarotene + shRNA-slc10a2, bexarotene + slc10a2-overexpression respectively, H1299 cells without treatment as control. (B) The apoptosis of H1299 cells treated with bexarotene, bexarotene + shRNA-slc10a2, bexarotene + slc10a2-overexpression respectively, H1299 cells without treatment as control. All experiments were repeated 3 times. (TIFF 739 kb)

Additional file 2: **Figure S2.** (A) The expression of apoptotic related genes Bcl-2, cyclin D1, c-FLIP, caspase 3, caspase 7 in H1299 cells treated with bexarotene, overexpressed slc10a2 in combination with bexarotene, slc10a2-shRNA in combination with bexarotene respectively. (B) The expression of tumor suppressor genes PTEN, P21, P53, LKB1, TSC2 in H1299 cells treated with bexarotene, overexpressed slc10a2 in combination with bexarotene, slc10a2-shRNA in combination with bexarotene respectively, H1299 cells without any treatment as control group. All experiments were repeated 3 times. (TIFF 516 kb)

Additional file 3: **Figure S3.** (A) The expression of apoptotic related genes Bcl-2, cyclin D1, c-FLIP, caspase 3, caspase 7 in H1299 cells when treated with bexarotene, bexarotene in combination with GW9662 respectively. (B) The expression of apoptotic related genes Bcl-2, cyclin D1, c-FLIP, caspase 3, caspase 7 in slc10a2 overexpressed H1299 cells when treated with bexarotene, bexarotene in combination with GW9662 respectively. (C) The expression of tumor suppressor genes PTEN, P21, P53, LKB1, TSC2 in H1299 cells when treated with bexarotene, bexarotene in combination with GW9662 respectively. (D) The expression of tumor suppressor genes PTEN, P21, P53, LKB1, TSC2 in slc10a2 overexpressed H1299 cells when treated with bexarotene, bexarotene in combination with GW9662 respectively. H1299 cells without any treatment as control group. All experiments were repeated 3 times. (TIFF 882 kb)

Additional file 4: **Figure S4.** The expression of slc10a2 in A549 cells treated with 1 mM, 5 mM, 1 0 mM bexarotene respectively, A549 cell without treatment as control. (TIFF 68 kb)

## Abbreviations

ANOVA: One-way analysis of variance
CCK-8: Cell Counting Kit-8
HDL: High-density lipoprotein
NSCLC: Non-small cell lung cancer
PPAR: Peroxisome proliferator-activated receptor
PPRE: Peroxisome proliferator responsive element
RXRs: Retinoid X receptors
TZD: Thiazolidinedione
