# Supplementary figures and images for: Bexarotene inhibits the viability of non-small cell lung cancer cells via slc10a2/PPARγ/PTEN/mTOR signaling pathway
Source: BMC Cancer. 2018 Apr 11;18:407. doi: 10.1186/s12885-018-4224-x (PMC5896077; doi:10.1186/s12885-018-4224-x)

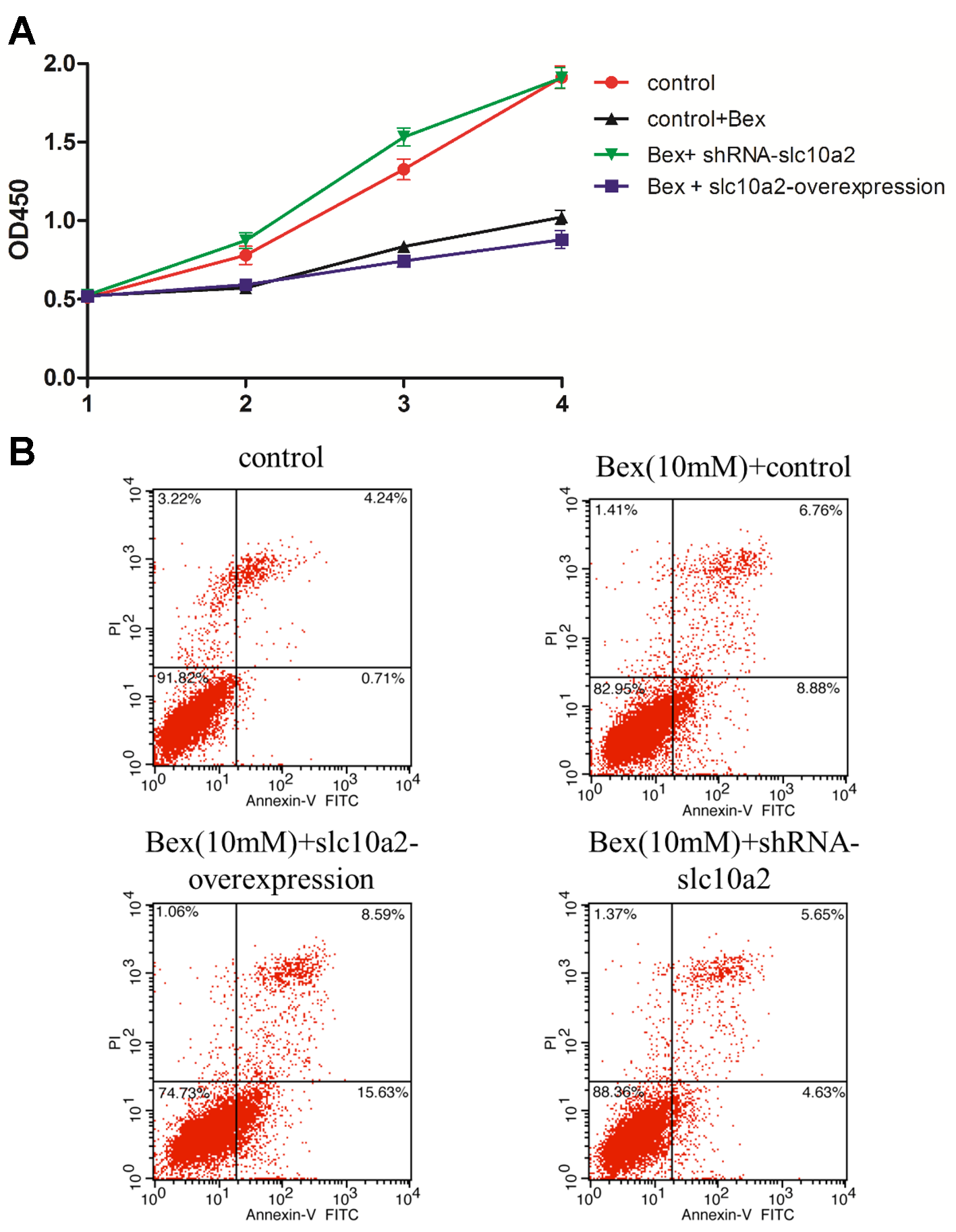

Supplement: Supplementary file 1 — Figure S1. (A) The proliferation of H1299 cells treated with bexarotene, bexarotene + shRNA-slc10a2, bexarotene + slc10a2-overexpression respectively, H1299 cells without treatment as control. (B) The apoptosis of H1299 cells treated with bexarotene, bexarotene + shRNA-slc10a2, bexarotene + slc10a2-overexpression respectively, H1299 cells without treatment as control. All experiments were repeated 3 times. (TIFF 739 kb) [file 12885_2018_4224_MOESM1_ESM.tiff]

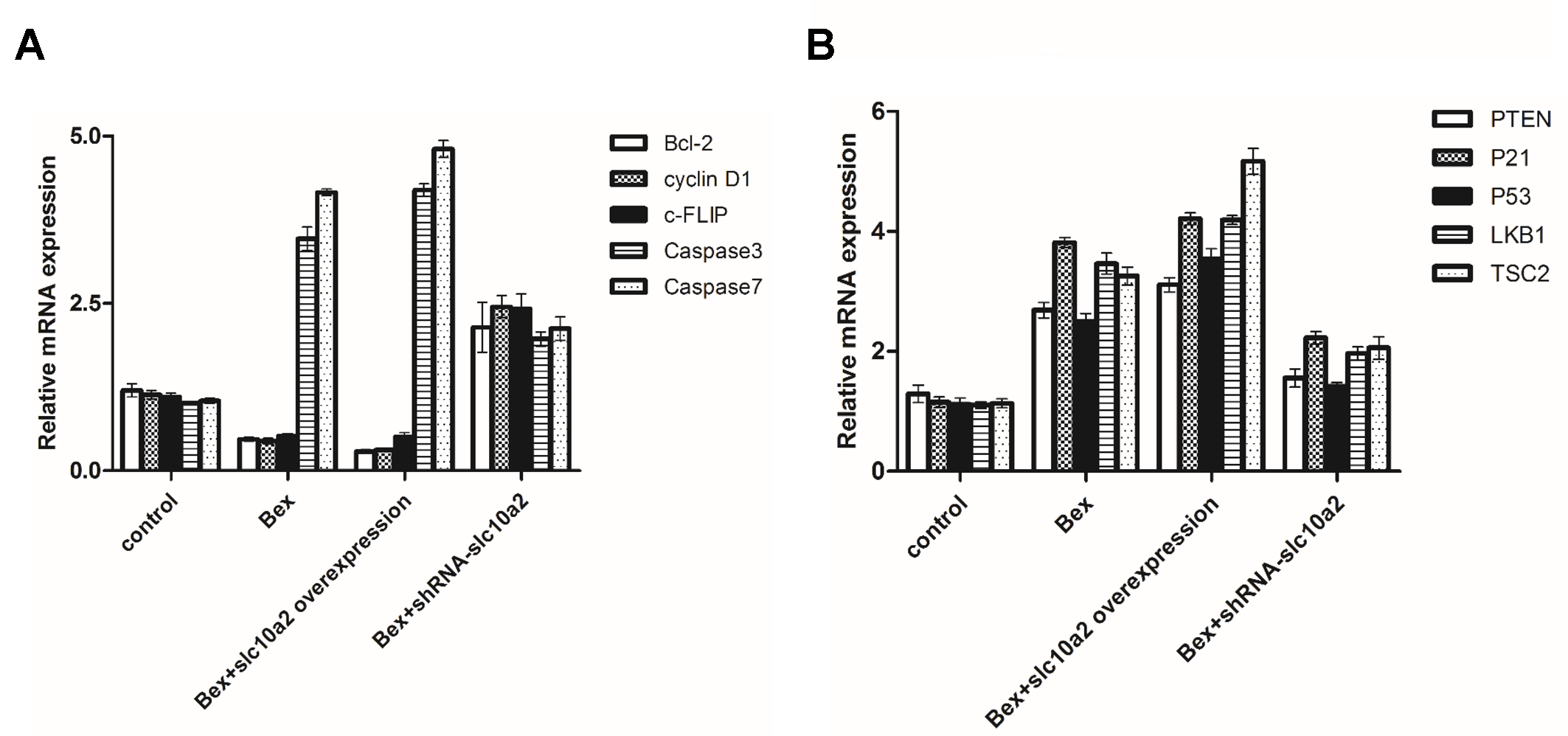

Supplement: Supplementary file 2 — Figure S2. (A) The expression of apoptotic related genes Bcl-2, cyclin D1, c-FLIP, caspase 3, caspase 7 in H1299 cells treated with bexarotene, overexpressed slc10a2 in combination with bexarotene, slc10a2-shRNA in combination with bexarotene respectively. (B) The expression of tumor suppressor genes PTEN, P21, P53, LKB1, TSC2 in H1299 cells treated with bexarotene, overexpressed slc10a2 in combination with bexarotene, slc10a2-shRNA in combination with bexarotene respectively, H1299 cells without any treatment as control group. All experiments were repeated 3 times. (TIFF 516 kb) [file 12885_2018_4224_MOESM2_ESM.tif]

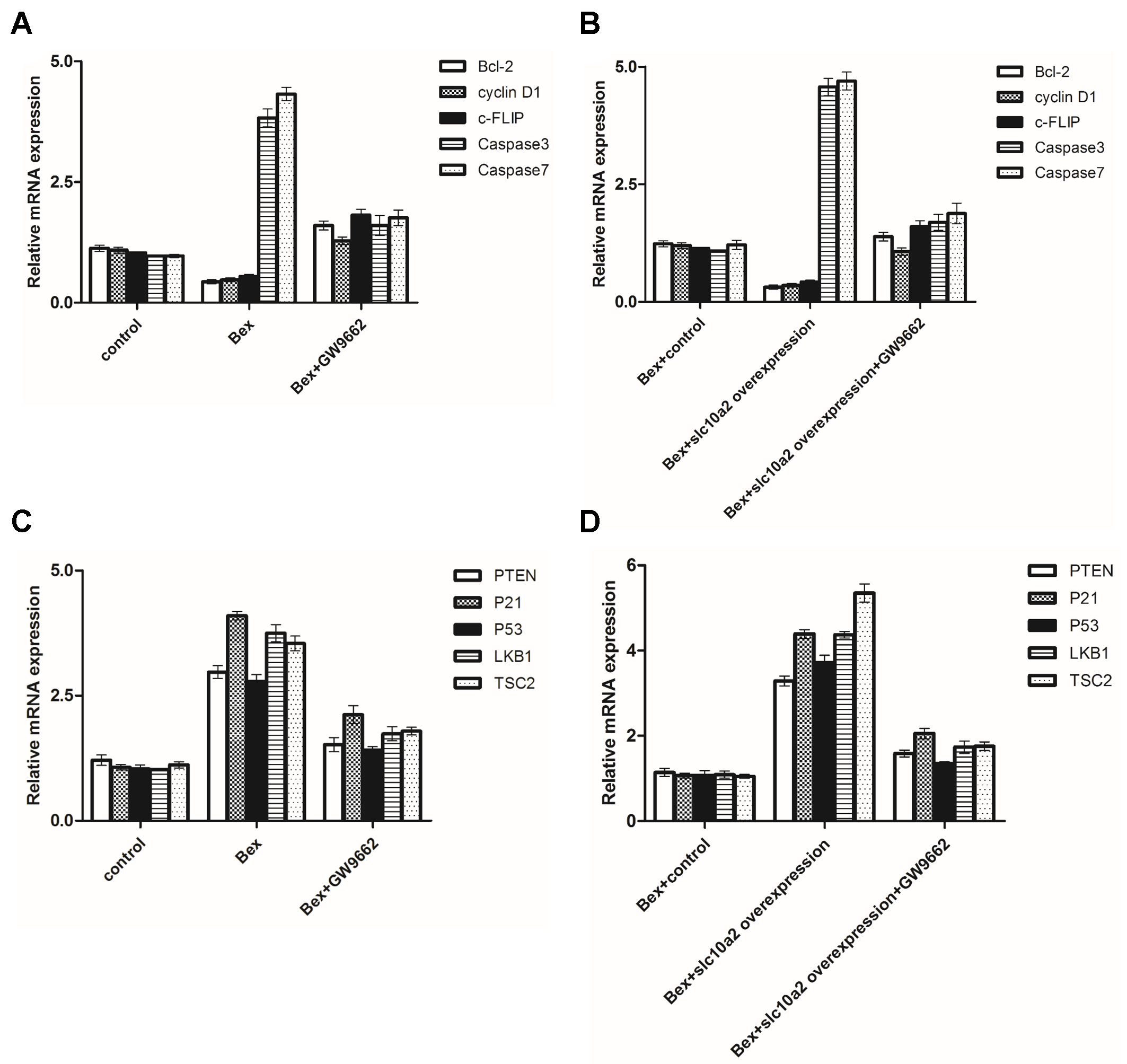

Supplement: Supplementary file 3 — Figure S3. (A) The expression of apoptotic related genes Bcl-2, cyclin D1, c-FLIP, caspase 3, caspase 7 in H1299 cells when treated with bexarotene, bexarotene in combination with GW9662 respectively. (B) The expression of apoptotic related genes Bcl-2, cyclin D1, c-FLIP, caspase 3, caspase 7 in slc10a2 overexpressed H1299 cells when treated with bexarotene, bexarotene in combination with GW9662 respectively. (C) The expression of tumor suppressor genes PTEN, P21, P53, LKB1, TSC2 in H1299 cells when treated with bexarotene, bexarotene in combination with GW9662 respectively. (D) The expression of tumor suppressor genes PTEN, P21, P53, LKB1, TSC2 in slc10a2 overexpressed H1299 cells when treated with bexarotene, bexarotene in combination with GW9662 respectively. H1299 cells without any treatment as control group. All experiments were repeated 3 times. (TIFF 882 kb) [file 12885_2018_4224_MOESM3_ESM.tif]

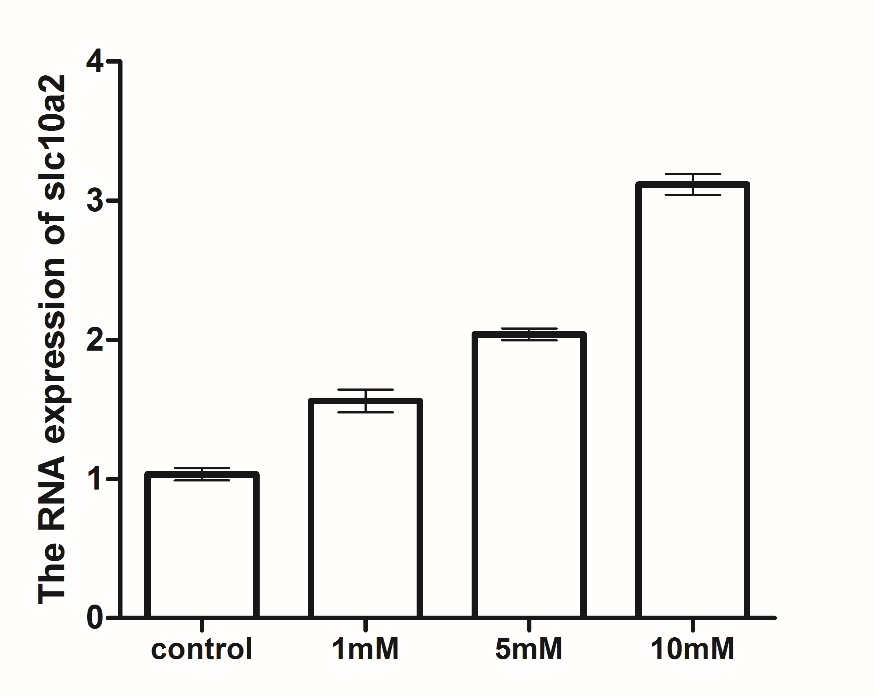

Supplement: Supplementary file 4 — Figure S4. The expression of slc10a2 in A549 cells treated with 1 mM, 5 mM, 1 0 mM bexarotene respectively, A549 cell without treatment as control. (TIFF 68 kb) [file 12885_2018_4224_MOESM4_ESM.tif]
